# Comparative kinematical analyses of Venus flytrap (*Dionaea muscipula*) snap traps

**DOI:** 10.3762/bjnano.7.59

**Published:** 2016-05-04

**Authors:** Simon Poppinga, Tim Kampowski, Amélie Metzger, Olga Speck, Thomas Speck

**Affiliations:** 1 Plant Biomechanics Group, Botanic Garden, University of Freiburg, Schänzlestraße 1, 79104 Freiburg, Germany; 2 Freiburg Materials Research Center (FMF), University of Freiburg, Stefan-Meier-Straße 21, 79104 Freiburg, Germany; 3 Freiburg Centre for Interactive Materials and Bio-Inspired Technologies (FIT), Georges-Köhler-Allee 105, 79110 Freiburg, Germany

**Keywords:** biomechanics, carnivorous plant, Droseraceae, fast plant movement, functional morphology

## Abstract

Although the Venus flytrap (*Dionaea muscipula*) can be considered as one of the most extensively investigated carnivorous plants, knowledge is still scarce about diversity of the snap-trap motion, the functionality of snap traps under varying environmental conditions, and their opening motion. By conducting simple snap-trap closure experiments in air and under water, we present striking evidence that adult *Dionaea* snaps similarly fast in aerial and submersed states and, hence, is potentially able to gain nutrients from fast aquatic prey during seasonal inundation. We reveal three snapping modes of adult traps, all incorporating snap buckling, and show that millimeter-sized, much slower seedling traps do not yet incorporate such elastic instabilities. Moreover, opening kinematics of young and adult *Dionaea* snap traps reveal that reverse snap buckling is not performed, corroborating the assumption that growth takes place on certain trap lobe regions. Our findings are discussed in an evolutionary, biomechanical, functional–morphological and biomimetic context.

## Introduction

**Figure 1:**
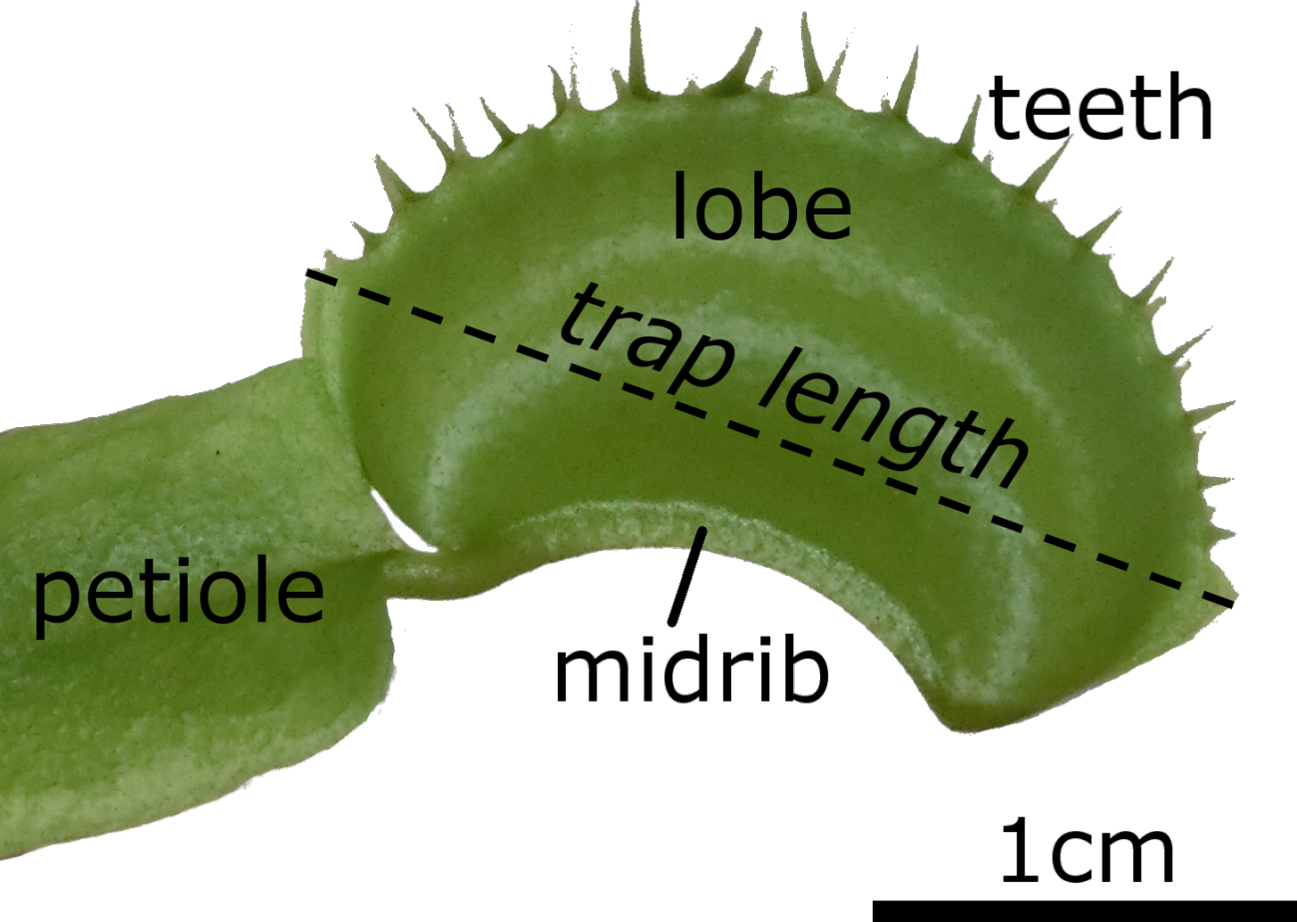
A closed *Dionaea muscipula* trap. Petiole and leaf blade serve the function of photosynthesis. In addition, the leaf blade is highly modified and contains a midrib which connects the two trap lobes. On the upper margins of the lobes, several “teeth” are located which interlock in the closed state of the trap. Trap lengths as presented in this article were measured as indicated.

The terrestrial Venus flytrap (*Dionaea muscipula*) is certainly the most iconic carnivorous plant [1–3], but the spectacular movement of its snap traps (Figure 1) is not yet fully understood. After reception of mechanical stimuli by prey on trigger hairs, the two trap lobes begin to move towards each other. This initial motion is mainly driven by active hydraulic actuation (i.e., turgor-induced cell deformation) [4–5], but a relaxation of mechanically pre-stressed mesophyll cells could also play a supporting role [6–7]. The concave lobes (as seen from the outside) store elastic energy during the initial motion, which is suddenly released when they flip to a convex curvature [8]. This second motion step, the snap-buckling process, greatly enhances the overall movement speed of the relatively large traps (ca. 2 cm in length), so that also fast prey can be caught [9]. After the relatively slow initial and rapid second motion step, the trap closes further but much slower owing to a poroelastically dampened motion of the hydrated lobes [8]. For digestion of prey, the trap forms a firmly sealed digestion chamber [2] that acts similar to an animal stomach.

Knowledge about the functionality of the traps under various, naturally occurring environmental conditions and in the different developmental stages of the plant is scarce at best. *Dionaea* grows in habitats that become seasonally inundated [10–11], can reportedly grow in a submersed state for months and is also capable of capturing aquatic animals, e.g., newts [12]. Detailed investigations regarding these potentially coincidental captures do not exist, and the question arises whether the traps function reliably under water. Conceivably, the denser surrounding medium (water) dampens the snapping motion, and water and prey potentially may become flushed out of the trap during snapping [13–14]. Moreover, *Dionaea* seedlings already possess a carnivorous habit [15], but nothing is known about trap closure kinematics in such an early stage of growth. What is more, although it is generally known that growth processes lead to trap opening [2], the lobe movement during this process and possible implications for the underlying opening mechanics has not yet been investigated.

For shedding some light on the above mentioned questions, we tested the snapping performance in terms of closure duration for submersed adult traps compared to traps snapping in air, and analyzed if trap closure leads to considerable water displacement out of the trap during closure. We additionally characterized different types of snapping modes which we observed during the above tests, investigated the snapping motions of seedlings and, furthermore, the opening kinematics of a young and an adult snap trap.

## Results

Raw data, analytical procedures and detailed results are presented in Supporting Information File 1 (raw data), Supporting Information File 2 (statistical analyses for the comparative snapping experiment) and Supporting Information File 3 (statistical analyses for the seedling snapping experiments).

### Snapping modes and trap performance under water

**Figure 2:**
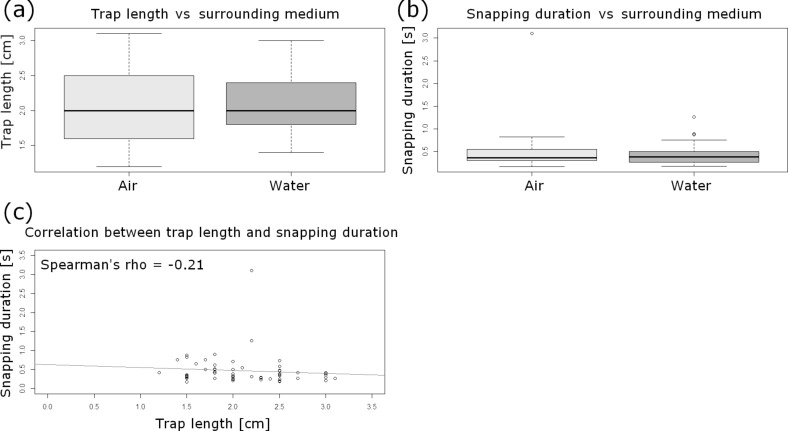
Statistical analyses of the comparative air/water snapping experiment. (a) Boxplot comparison of trap lengths in air and under water. The sample sizes for each surrounding medium is *n* = 30. The trap lengths are not significantly different between the different surrounding media (Wilcoxon rank sum test, *W* = 487.5; *p* > 0.05). (b) Boxplot comparison of snapping durations in air and under water. The sample sizes for each surrounding medium is *n* = 30. The snapping durations are not significantly different between the different surrounding media (Wilcoxon rank sum test, *W* = 472; *p* > 0.05). (c) Snapping durations do not correlate significantly with the trap lengths (Spearman‘s *rho* = −0.21). The regression line is indicated.

Testing of a set of general assumptions during the statistical analyses revealed that the underlying dataset of snapping events (air/water) (Supporting Information File 1) is independent, not normally distributed (Shapiro–Wilk test) and homoscedastic (Levene test, *car* package). Additionally, the descriptive statistics confirmed these characteristics. The trap lengths are not significantly different between the traps analyzed in the different surrounding media (Wilcoxon rank sum test, *W* = 487.5; *p* > 0.05) (Figure 2a). The median trap length is 2 cm (IQR: 0.73 cm; min: 1.2 cm; max: 3.1 cm) (*n* = 60). The snapping durations are not significantly different between the traps analyzed in the different surrounding media (Wilcoxon rank sum test, *W* = 472; *p* > 0.05) (Figure 2b) and do not correlate with the trap lengths (Spearman‘s *rho* = −0.21) (Figure 2c). The median snapping duration is 0.37 s (IQR: 0.23 s; min: 0.17 s; max: 3.1 s) (*n* = 60). Since neither trap lengths nor snapping durations differ significantly between the given surrounding media, we were able to pool the data.

**Figure 3:**
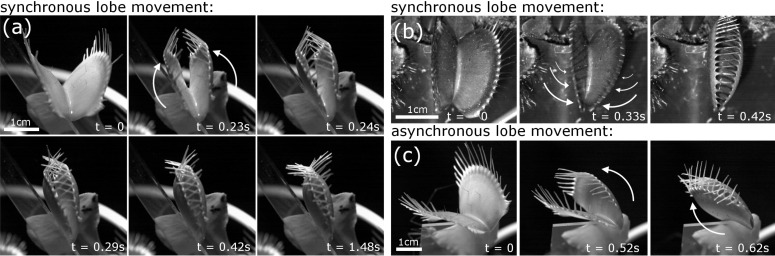
Snapping modes of Venus flytrap. (a) Synchronous lobe movements either lead to a sudden curvature inversion of both trap lobes (“normal” snapping), or (b) to a snap buckling beginning at the apical part of the trap and progressing towards the basal part. (c) In asynchronous trap lobes, one of the lobes moves first. Time scales are indicated, arrows depict lobe movement. At *t* = 0.29 s in (a), the trap is in the state defined as the closed state in this article (see Experimental section). Afterwards, the poroelastically dampened closure motion proceeds, but much slower (see timescales).

We recognized the following snapping modes among the 60 traps tested in the comparative air/water analysis: Synchronously moving trap lobes (found in 39 traps) either perform a “normal” snapping, with a sudden snap buckling of the two lobes (in 38 traps) (Figure 3a, Supporting Information File 4), or a progressive snapping, with the closing motion and snap buckling beginning at the apical part of the trap and progressing towards the basal part (only in one trap) (Figure 3b, Supporting Information File 5). The lobes of the other 21 traps possessed strikingly asynchronously moving lobes (Figure 3c); either the triggered lobe moved first (12 traps) (Supporting Information File 6), or the non-triggered lobe (9 traps) (Supporting Information File 7). Snapping modes are independent from the surrounding medium (air/water) (Fisher’s exact test, *p* > 0.05) (Supporting Information File 2).

**Figure 4:**
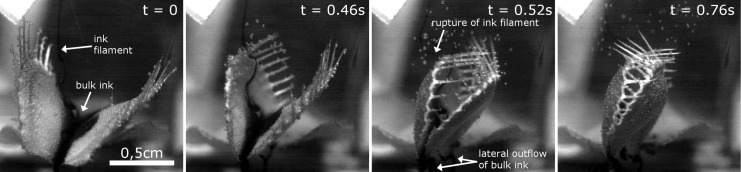
Snapping of a trap under water. Time scale is indicated. An ink filament reaching into the trap with the ink drop is visible. During snapping, no noticeable distortion of the filament, but an outflow of bulk ink out of small gaps at the lateral trap parts is visible. The trap lobes move asynchronously, whereby the triggered lobe (the left lobe in the images) moves first. Images are from Supporting Information File 8.

Ink drops deposited into the nine submersed traps were not subject to considerable outflows during snapping, which would have indicated a theoretical flushing out of prey. The trap lobes perform a motion similar to a clasping movement around the water body and the ink drop inside the trap, which is apparently due to the three-dimensional bending deformation of the lobes. We observed asynchronous as well as “normal” snapping motions in the nine traps. In one video (Supporting Information File 8, Figure 4), a remaining ink thread extending from the syringe is clearly visible. This thread is not distorted during the closure motion until it becomes ruptured by a trap lobe. This indicates that the water body in the trap lumen indeed is rather undisturbed by the movement of the closing lobes. Even after trap closure, the ruptured thread is visible inside the trap and apparently undistorted. Nonetheless, an outflow of water and ink through narrow gaps at the apical and basal ends of the trap is visible.

### Comparative kinematics of seedling and adult traps

**Figure 5:**
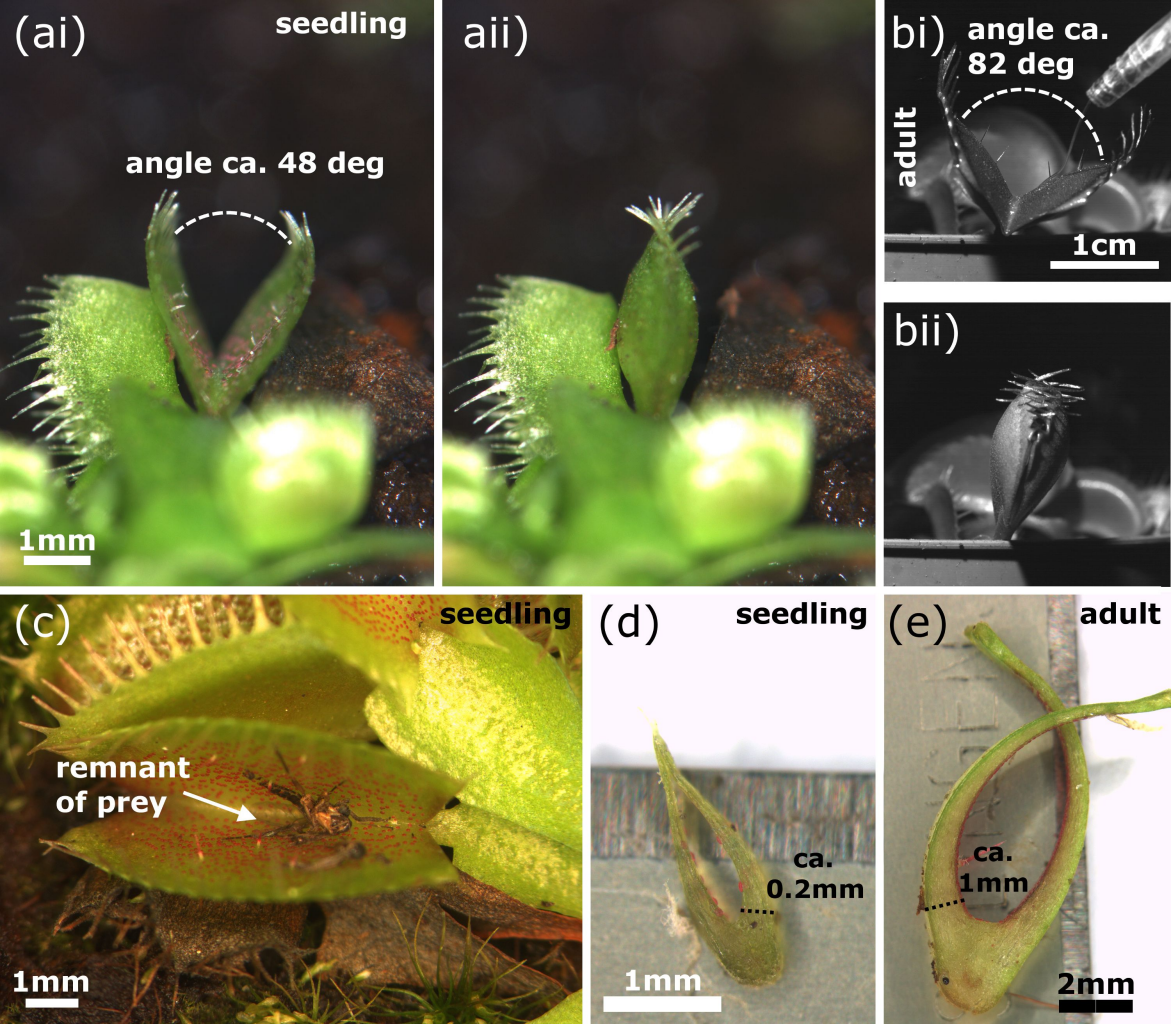
Seedling and adult traps. (a) A seedling trap in the open and closed state, the opening angle is indicated. (b) An adult trap in the open and closed state, the opening angle is indicated. (c) Seedlings cultivated in the Botanic Garden Freiburg often showed arthropod remnants inside the traps, indicating successful prey capture. (d) Section of a seedling trap. (e) Section of an adult trap. (d) and (e) were used for approximations of lobe thicknesses required for calculations in the discussion.

All traps of the analyzed seedlings showed synchronous lobe closure movements. Seedling traps either do not close completely, with the motion stopping when the marginal teeth are in contact with each other, or perform a “normal” closing motion as described above (Figure 5). The angle between the open seedling trap lobes is noticeably smaller as in adult traps. We measured ca. 48° in one seedling and ca. 82° in one adult trap (Figure 5a,b).

**Figure 6:**
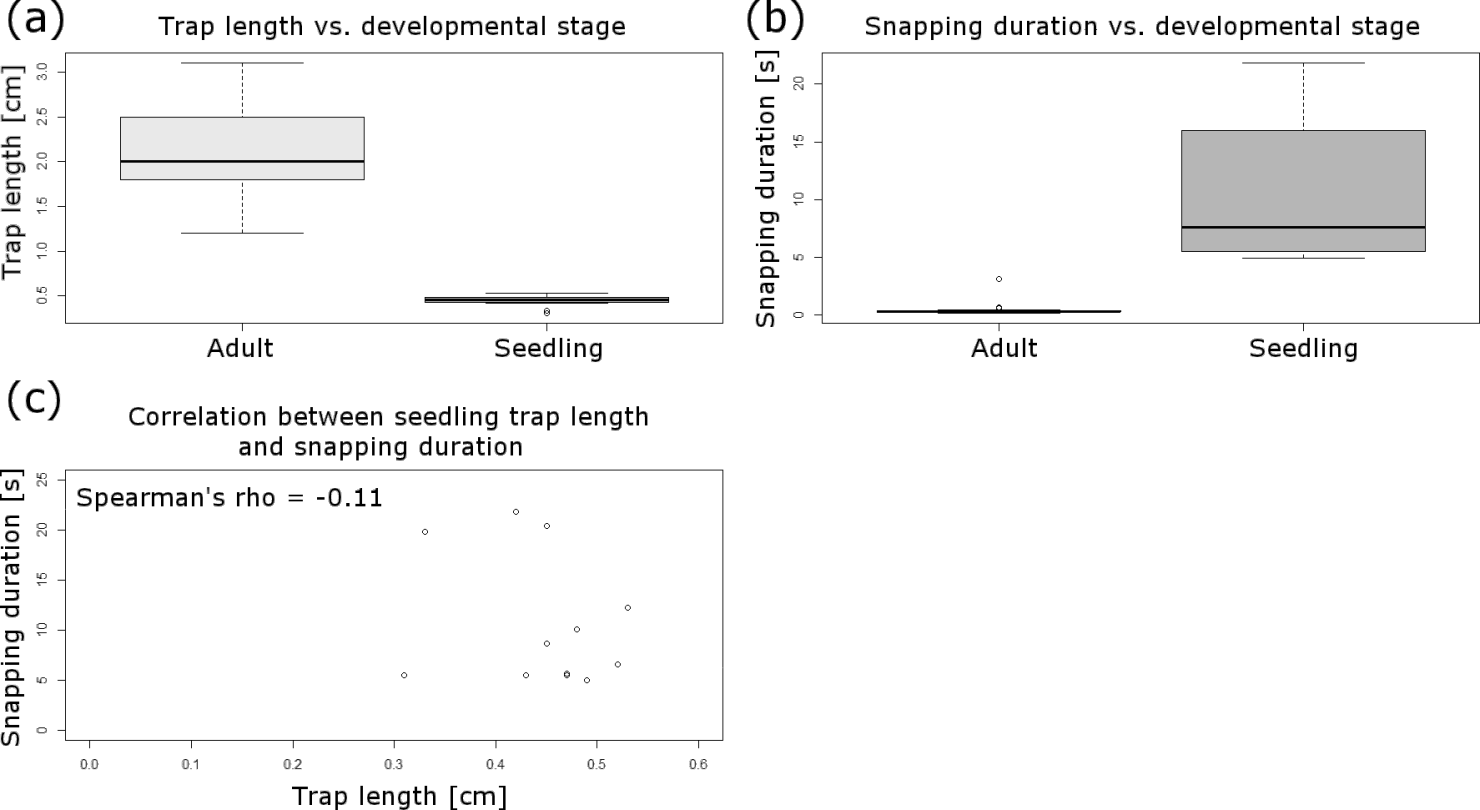
Statistical analyses of the comparative seedling trap/adult trap snapping experiment. (a) Boxplot comparison of trap lengths in different developmental stages of the Venus flytrap. The sample size for adult traps is *n* = 21 (see Experimental section), for seedlings *n* = 12. The trap lengths are highly significantly different between the two growth stages. (b) Boxplot comparison of snapping durations in different developmental stages of the Venus flytrap. The sample size for adult traps is *n* = 21 (see Experimental section), for seedlings *n* = 12. The snapping durations for adult traps are highly significantly shorter than for seedling traps. (c) Snapping durations do not correlate with the trap lengths in seedlings (Spearman‘s *rho* = −0.21).

Testing of a set of general assumptions during the statistical analyses revealed that the underlying dataset (Supporting Information File 1) of seedling trap lengths is normally distributed, whereas snapping durations are not normally distributed, and both sets are heteroscedastic. The median length of seedling traps is 0.46 cm (IQR: 0.06 cm; min: 0.31 cm; max: 0.53 cm) (*n* = 12), which is – not surprisingly – highly significantly different from the trap lengths found for adult traps (Wilcoxon rank sum test, *W* = 252; *p* < 0.001) (Figure 6a). The median snapping duration of seedling traps is 7.63 s (IQR: 8.61 s; min: 4.96 s; max: 21.82 s) (*n* = 12), which is highly significantly longer than the closing durations found for adult traps (Wilcoxon rank sum test, *W* = 0; *p* < 0.001) (Figure 6b), and also showed no correlation with the trap lengths (Spearman‘s *rho* = −0.11) (Figure 6c).

**Figure 7:**
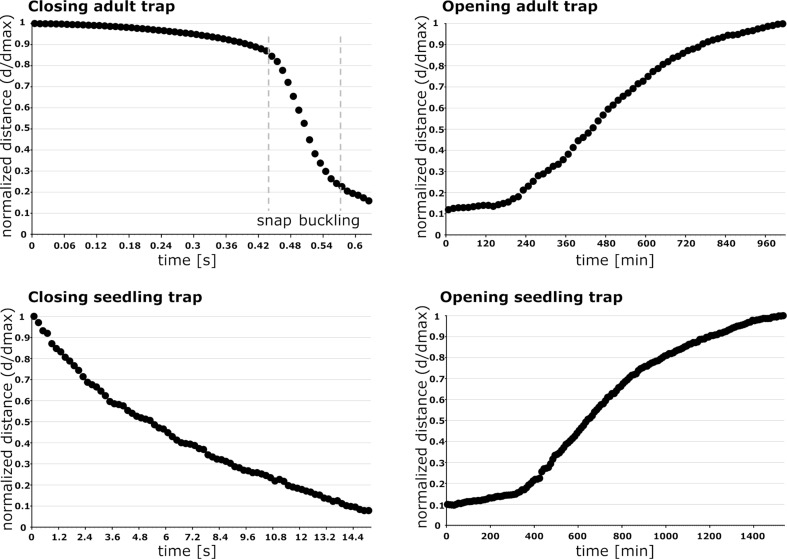
Comparative kinematics of closing and opening motions in adult and seedling traps. Note the different time scales indicated. The adult trap closes very rapidly and performs a sudden geometrical change (snap buckling, indicated by dashed grey lines), whereas the much slower closing of the seedling trap is a very continuous process without any noticeable acceleration. Opening traps also move very continuously, indicating that no reverse snap-buckling takes place either in the adult traps or in the seedling traps. The normalized distance d* is calculated as ratio of *d*, the remaining distance between the lobes measured for various phases of closure, over *d*_max_, the distance between the lobes in the fully open trap.

The seedling trap motions are very continuous and not characterized by the otherwise typical movement steps differing in speed observed in adult traps (slow, fast, slow) (Figure 7). Additionally, no inversion of lobe curvature is visible (Supporting Information File 9). The adult trap analyzed in these experiments closed much faster within ca. 0.6 s (which is in general agreement with the snapping durations for adult snap traps described above) and shows a rapid intermediate lobe distance decrease, which can be attributed to the fast shape change caused by snap buckling. Trap opening took ca. 24 h in the seedling (Supporting Information File 10) and 16 h in the adult trap (Supporting Information File 11). In both traps, it represents a continuous process without any sudden acceleration of the lobes (Figure 7).

## Discussion

Our analyses indicate that several snapping modes exist in adult *Dionaea* traps (Figure 3). As we did not perform repetitive experiments with the same traps, we cannot give an answer to the question if these phenomena are recurrent, i.e., if they are morphologically and/or physiologically predetermined for a given trap. In what way the modes influence (i.e., favor or hinder) prey capture can neither be concluded from this study, but we can assume (according to the short trap closure durations measured for all snapping modes) that *Dionaea* is theoretically capable of capturing prey with any mode. Also, it remains to be answered how these diverging post-stimulatory mechanical answers are evoked. Probably, differences in the processes of stimulus signal transduction, reception and processing [16–19], as well as differences in trap lobe anatomy, mechanical properties or the general vigor of the plant influence the process and the mode of snapping [2,20–23]. Our observations on the different modes of trap closing could be of potential interest for biomimetic approaches [24] where fast and large-scale deformation of thin shells as well as principles for generation, storage and release of elastic energy are important.

As adult traps close similarly fast under water as they do in air (Figure 2b), and because of the fact that no considerable water displacement out of the trap is visible during this process (Figure 4), the Venus flytrap is without doubt capable of capturing fast aquatic prey. We consider it as unlikely that prey could escape through the small lateral gaps of the closed trap which are visible in Figure 4 and in Supporting Information File 8. Detailed future analyses should take into account using tracer particles, several cameras and particle image velocimetry (PIV) methods for tracking the water displacement. The question if trapping events under water occur only occasionally and by coincidence is also a matter for possible future studies. The factor “prey attraction” should additionally be analyzed, i.e., to what extent it plays a role for the submersed plant. Moreover, future studies could also tackle the question if *Dionaea* is able to perform prey digestion under water, i.e., if the digestion chamber is sealed watertight.

The sister species to *D. muscipula* is the aquatic Waterwheel plant (*Aldrovanda vesiculosa*) [25–26]. *Aldrovanda* features snap traps under water that are similarly fast as the aerial traps of *Dionaea*, but which, in contrast, are small enough (ca. 4–5 mm in length) to move purely hydraulically. The much smaller *Aldrovanda* trap can be actuated hydraulically ca. 100 times more rapidly than the *Dionaea* trap [9]. Triggering by prey entails turgor changes in motor cells [27–31] leading to a bending of a midrib kinematically coupled to the trap lobes, which simultaneously close without performing curvature inversions [13,27,32]. Hence, the *Dionaea* trap consists of two independent, active kinematical elements (the lobes, each performing hydraulic motion and snap buckling), whereas the *Aldrovanda* trap consist of one kinematical element, the midrib, which actively bends and couples the undeformed lobes. We assume that the asynchronous *Dionaea* snapping as observed and described above is relevantly based on this division into two independent kinematical elements, and we consider it as unlikely that such a mode also occurs in *Aldrovanda* (which, however, remains to be investigated experimentally).

Due to a lack of fossilized intermediate forms, the question of how snap traps evolved is still a matter of debate. Gibson and Waller [33] argue that selection to catch and retain large prey favored evolution of snap traps in general, but after Hutchens and Luken [34], *Dionaea* prey capture is rather opportunistic than selective. Poppinga and Joyeux [13] have hypothesized that the different snap-trap mechanics are adaptations to the respective life form (terrestrial vs aquatic). Hence, the *Aldrovanda* mechanism could be an optimized means to reliably capture prey under water, whereas the *Dionaea* trap snaps shut rapidly on land. From our comparative study (air/water) we can conclude that the *Dionaea* trap mechanism (hydraulics plus elastic relaxation of a comparably large thin shell) is presumably not an adaptation to the terrestrial lifeform in regard to mere trap functioning, because the same mechanism works also well under water. In return, it would be interesting to perform a study comparable to the present one in which the *Aldrovanda* trap snaps in air. Owing to the small size of the *Aldrovanda* trap and due to the lower density of the surrounding medium, we may speculate that snapping in air is presumably of comparable (or even higher) speed than under water. So why do *Aldrovanda* and *Dionaea* possess two different trap closure mechanics and does there exist a selective advantage for one of the snapping modes in air or under water? Probably, the selection to catch and retain large prey [33] in combination with the ability to catch a broad range of prey of different sizes [34] favored the evolution of the *Dionaea* trap, whereas *Aldrovanda* relies on capture of abundant and small zooplankton [35–36].

*Dionaea* seedlings capture their prey with millimeter-sized traps (Figure 5). The closure durations measured are much higher than in the similarly small *Aldrovanda* traps and in adult *Dionaea* traps (Figure 6b). This presumably indicates that much slower prey is being caught in nature. In traps of our cultivated seedlings we often observed remnants of arthropods (Figure 5c), indicating successful prey capture and digestion as investigated in detail by Hatcher and Hart [15]. The angle between the lobes of seedling traps is small (Figure 5a), which is probably a structural requirement to achieve “reasonable” trap closure durations with (relatively) slow movement speeds.

Based on the snapping durations measured and on our kinematical analyses (Figure 7) we can now assume that snap buckling as a motion speed boost is not (or, to a much lesser degree) implemented as a feature already in young traps. It remains to be investigated if this is morphologically manifested in a lesser lobe curvature and/or lesser lobe thickness. Both parameters are known to strongly influence the snap-buckling behavior [8]. For calculating the theoretical speed of hydraulic actuation according to Skotheim and Mahadevan [9], we measured the approximate lobe thicknesses from photographs of seedling and adult traps crosscut in their mid-planes (Figure 5d,e). The thicknesses were measured at the bottom parts of the trap lobes where Forterre et al. [8] indicate the strongest lobe surface extensions caused by the hydraulically actuated movement. By approximation of a seedling trap lobe thickness of *L*_s_ ≈ 200 µm we see that the theoretical timescale for water displacement, the poroelastic time τ_ps_ ≈ 0.064 s, is well below the fastest snapping duration measured (*τ*_s_ = 4.96 s). This allows us to speculate that traps of Venus flytrap seedlings are indeed actuated mainly or exclusively hydraulically and that the additional feature of snap buckling probably only appears when “size matters”, i.e., in later developmental stages when the traps are too large to move fast and to become actuated mainly hydraulically. For lobes of adult traps with the typical thickness *L*_a1_ ≈ 500 µm [9], respectively with the measured thickness *L*_a2_ ≈ 1 mm, we have *τ*_pa1_ ≈ 0.4 s [9] and *τ*_pa2_ ≈ 1.6 s, which both are well above the measured fastest snapping duration (*τ*_a_ = 0.17 s). Presumably, the *Aldrovanda* trap is much faster as a *Dionaea* seedling trap owing to the kinematic amplification mechanism, which enables large-scale deformation with a minute initial, hydraulically evoked deformation of the midrib [13,32].

The opening of the analyzed adult trap and seedling trap is a very slow process (adult: ca. 16 h; seedling: ca. 24 h). The motions are continuous and without any noticeable sudden acceleration (Figure 7). We speculate that growth [2] takes place on certain lobe region(s) and that the mechanically difficult and, hence, energetically costly (or even impossible?) inversion of lobe curvature by reverse snap buckling is avoided in adult traps. As the process of cell elongation during growth is naturally limited, the traps die once a critical length has been reached (after 3–12 snapping actions, [11]). Theoretically, traps of seedlings could reopen by simply reversing the processes involved in hydraulic snapping without a growth-based increase in size, which might be beneficial for repetitive prey capture and survival in such a young developmental stage.

Our results showing that the snapping duration in adult traps does not correlate with trap length (Figure 2c) seem to contradict the hypothesis formulated by Forterre et al. [8], who postulate that larger traps should have higher snapping speeds. This is based on the finding that the snap-buckling behavior depends on the dimensionless parameter α = *W*^4^·κ^2^/*h*^2^, with *W* being the size of the leaf, κ the mean curvature of the trap lobes, and *h* the leaf thickness. As we did not measure *W* and κ in our study and because the traps investigated are more or less similar in size, our results have to be regarded with caution, and further comparative studies should take the mentioned parameters and a larger variety of trap sizes into consideration. Figure 2b shows that there are no conspicuous variations in terms of measured closure durations between the traps tested underwater (15 °C) and in air (20–25 °C), which indicates that movement speed is not or only to a little degree influenced by temperature. Nonetheless, because the duration of the overall trap motion is ultimately dictated by the flow of interstitial water through the lobes, future comparative snapping experiments should be performed in temperature-constant chambers to exclude even small temperature-dependent physiological differences.

As a conclusion it can be said that the Venus flytrap has evolved a remarkable trapping system that functions as well in air as under water, and which can be considered as an optimized system for nutrient acquisition of a carnivorous plant growing in seasonally inundated habitats. Similar reports on carnivorous plants with traps functioning under different environmental conditions are, e.g., the resinous *Roridula* sticky traps [37] and the rainwater-dependent pitfall trapping systems in *Nepenthes* [38–39]. The *Dionaea* trap is not “only” a “simple” snap trap but possesses different snapping modes, movement mechanics and actuation principles, which greatly broadens our understanding of this (in)famous carnivore and opens up novel perspectives for future studies.

## Experimental

### Plant material

We analyzed healthy, well-watered and potted adult *D. muscipula* plants as well as half-year-old seedlings, all cultivated in a temperate greenhouse of the Botanic Garden Freiburg.

### General cinematographic analyses

For filming fast closure motions, traps were stimulated with a nylon thread on the trigger hairs of one lobe and recorded with a high-speed camera (Motion Scope Y4, Redlake, USA, recording speed 100 fps) in combination with a macro objective lens (Zeiss Makro-Planar T*2/100 mm ZF), a cold light source (techno light 270, Karl Storz GmbH & Co.KG, Tuttlingen) and by using the software Motion Studio x64 2.12.12.00 (IDT, USA). For filming the slow opening motions, the closed traps were recorded with a ColorView II camera (recording speed: 1 frame per 10 or per 15 min) mounted on a horizontally adjusted SZX9 stereo microscope and by using the cell^D 2.6 software (Olympus Corp., Tokyo, Japan). Trap sizes were measured after closure at their largest lateral dimensions either directly on the plant or from photographs by using Fiji/ImageJ 1.48r [40] (Figure 1).

### Comparison of snapping durations in air and under water and characterization of snapping modes

Traps were recorded in air (temperature in our lab ca. 20–25 °C, relative air humidity ca. 34.5–43.5%) and under water. For each trial we used 30 different traps from 30 different plants so that, in total, 60 traps from 60 plants were tested. For the experiments with submersed traps, the respective potted plants were consecutively placed in a 20 cm deep, small plastic tank filled with tap water (temperature ca. 15 °C). Warmer water (20–25 °C) was not used because it often led to undesired trap closure.

Video analyses were performed with Fiji/ImageJ as described above. Firstly, the snapping events were categorized whether the trap lobes close simultaneously or not. Traps with noticeably asynchronous lobes were subdivided into traps where the stimulated lobe moves first, and where the not-stimulated lobe moves first. From all snapping events the snapping durations were measured. For this purpose, we counted the number of video frames starting from the last frame where no motion is visible until the frame where the teeth of the lobe margins interlock in such a way that they extend to the other lobe’s margin (see Figure 2). We chose this point in time as the “closed state” because 1) the important and fast snap-buckling process is already finished, 2) in this state the interlocking teeth presumably effectively deter escape of prey by forming a mechanically strong mesh, 3) because the subsequent, final closure movement step is poroelastically dampened and very variable in duration and is also often influenced by teeth blocking the counterpart lobe.

Only smooth and failure-free motions were analyzed. In the cases where the motion of the lobes was asynchronous, we counted the frames from the beginning of the motion of the first lobe until both lobes have moved to the above described closed state.

We used the software GNU R 3.1.1 [41] including the additional packages “*car*” [42] and “*psych*” [43] for statistical analyses (Supporting Information File 2). We analyzed trap lengths and snapping durations for significant differences for traps tested under aerial or submersed conditions using Wilcoxon rank sum tests. The relationship between trap length and snapping duration was examined calculating Spearman’s rank correlation coefficient (*rho*), whereas the dependency between the snapping mode and the surrounding medium was studied using Fisher’s exact test.

### Qualitative analysis of water displacement during snapping under water

We carefully placed several ink drops (T10 blau, Lamy GmbH, Heidelberg-Wieblingen,) with a syringe onto the midribs of nine submerged traps, which were subsequently stimulated and recorded as described above. Video analyses were performed with Fiji/ImageJ.

### Comparative kinematical analyses of seedling and adult traps

Movements of 12 traps from different seedlings were recorded by using the high-speed-equipment described above and analyzed with Fiji/ImageJ. Trap closure durations in seedlings had to be calculated in another way as described for adult traps, because we found that the traps often do not close completely (see Results section). Therefore, we counted the frames from the beginning of the motion up to the frame were the motion stops completely.

For statistical analyses, we again used GNU R 3.1.1 including the same additional packages and procedures as described above (Supporting Information File 3). We used 21 adult traps with synchronously moving lobes from the above described comparative air/water analysis (see Results section) for comparison. We analyzed trap lengths and snapping durations of seedlings and the above mentioned adult traps for significant differences, and additionally analyzed the correlation between seedling trap length and snapping duration.

From the high-speed-videos of one additional seedling trap and from an additional adult trap we measured in each case the distance between the two lobes during snapping. Subsequently, we then calculated the normalized distance d* according to the formula


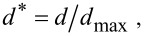


with *d* being the respective remaining distance measured and *d*_max_ the distance between the lobes in the fully open trap. One more additional seedling trap and one additional adult trap were recorded during opening as described above. From the resulting videos we also calculated the normalized distances d*.

## Supporting Information

File 1 MS Excel file with raw data.

File 2 Statistical analyses for the comparative air/under water snapping experiment.

File 3 Statistical analysis: Venus flytrap seedlings.

File 4 Movie file (.mov, MPEG-4 (Quick Time)) showing synchronous lobe movement (recording speed 100 fps, playback 20 fps).

File 5 Movie file (.mov, MPEG-4 (Quick Time)) showing progressive lobe movement (recording speed 100 fps, playback 20 fps).

File 6 Movie file (.mov, MPEG-4 (Quick Time)) showing asynchronous lobe movement (triggered lobe moves first) (recording speed 100 fps, playback 20 fps).

File 7 Movie file (.mov, MPEG-4 (Quick Time)) showing asynchronous lobe movement (non-triggered lobe moves first) (recording speed 100 fps, playback 20 fps).

File 8 Movie file (.mov, MPEG-4 (Quick Time)) showing trap closure under water. The ink drop inside the trap and the ink filament are visible (recording speed 100 fps, playback 20 fps).

File 9 Movie file (.mov, MPEG-4 (Quick Time)) showing closure of a seedling trap (recording speed 100 fps, playback 20 fps).

File 10 Movie file (.mov, MPEG-4 (Quick Time)) showing the opening of a seedling trap (recording speed 1 frame per 10 min, playback 20 fps).

File 11 Movie file (.mov, MPEG-4 (Quick Time)) showing the opening of an adult trap (recording speed 1 frame per 15 min, playback 20 fps).
