# Supplementary material for: Comparative kinematical analyses of Venus flytrap (Dionaea muscipula) snap traps
Source: Beilstein J Nanotechnol. 2016 May 4;7:664–74. doi: 10.3762/bjnano.7.59 (PMC4902084; doi:10.3762/bjnano.7.59)
Supplement: File 2 — Statistical analyses for the comparative air/under water snapping experiment. [file Beilstein_J_Nanotechnol-07-664-s002.pdf]

Supporting Information

for

Comparative kinematical analyses of Venus flytrap (*Dionaea muscipula*) snap traps

Simon Poppinga*[1,2], Tim Kampowski[1,2], Amélie Metzger[1], Olga Speck[1,2,3] and Thomas Speck[1,2,3]

Address: [1]Plant Biomechanics Group, Botanic Garden, University of Freiburg, Schänzlestrasse 1, 79104 Freiburg im Breisgau, Germany; [2]Freiburg Materials Research Center (FMF), University of Freiburg, Stefan-Meier-Straße 21, 79104 Freiburg im Breisgau, Germany and [3]Freiburg Center for Interactive Materials and Bio-Inspired Technologies (FIT), Georges-Köhler-Allee 105, 79110 Freiburg im Breisgau, Germany

Email: Simon Poppinga* - simon.poppinga@biologie.uni-freiburg.de

*Corresponding author

Statistical analyses for the comparative air/under water snapping experiment

Descriptive statistics

Descriptive statistics

Whole data set

Parameter	Trap length (cm)	Snapping duration (s)
Sample size (n)	60	60
Mean	2.08	0.47
SD	0.49	0.40
Median	2.00	0.37
IQR	0.73	0.23
Min	1.20	0.17
Max	3.10	3.10
Range	1.90	2.93

Descriptive statistics

Snapping in air

Parameter	Trap length (cm)	Snapping duration (s)
Sample size (n)	30	30
Mean	2.13	0.5
SD	0.56	0.52
Median	2.00	0.36
IQR	0.88	0.23
Min	1.20	0.17
Max	3.10	3.10
Range	1.90	2.93

Descriptive statistics

Snapping under water

Parameter	Trap length (cm)	Snapping duration (s)
Sample size (n)	30	30
Mean	2.04	0.44
SD	0.41	0.25
Median	2.00	0.38
IQR	0.58	0.24
Min	1.40	0.18
Max	3.00	1.26
Range	1.60	1.08

Descriptive statistics

Snapping modes:

Mode a: synchronous lobes

Mode b: asynchronous lobes: triggered lobe moves first

Mode c: asynchronous lobes: not-triggered lobe moves first

	Whole data set	In air	under water
**Mode a**	39 (65%)	21 (70%)	18 (60%)
**Mode b**	12 (20%)	7 (23%)	5 (17%)
**Mode c**	9 (15%)	2 (7%)	7 (23%)

Test of normal distribution

Test of normal distribution

Whole data set (GNU R 3.1.1; Shapiro-Wilk test; *shapiro.test()*-function; *stats*-package)

Trap length

No normal distribution
(W=0.9414; $p < 0.01$)

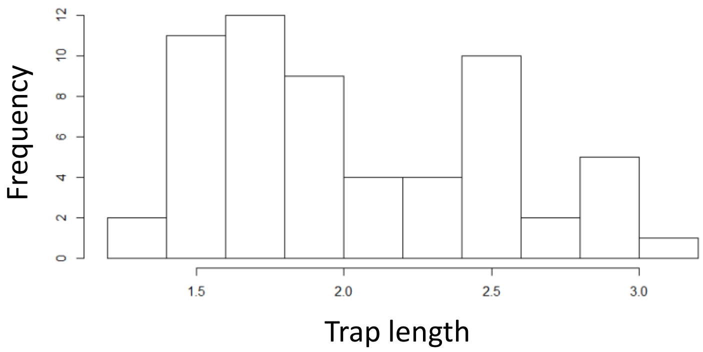

Snapping duration

No normal distribution
(W=0.5303; $p < 0.001$)

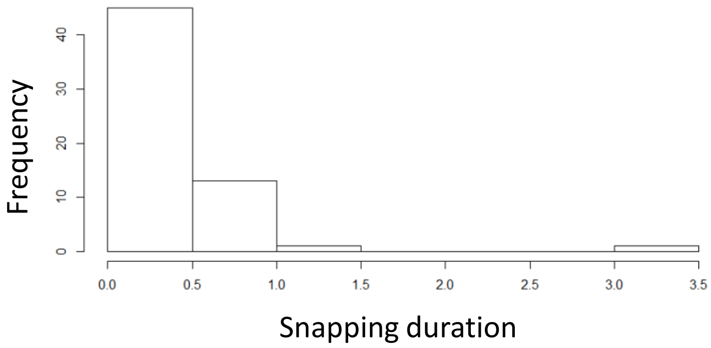

Test of normal distribution

In air (GNU R 3.1.1; Shapiro-Wilk test; *shapiro.test()*-function; *stats*-package)

Trap length

No normal distribution
(W=0.9283; $p < 0.05$)

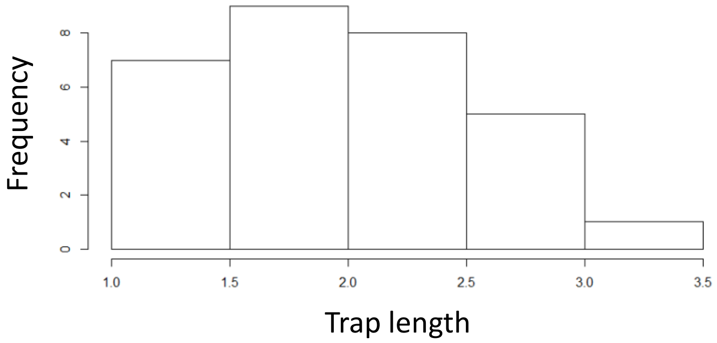

Snapping duration

No normal distribution
(W=0.4554; $p < 0.001$)

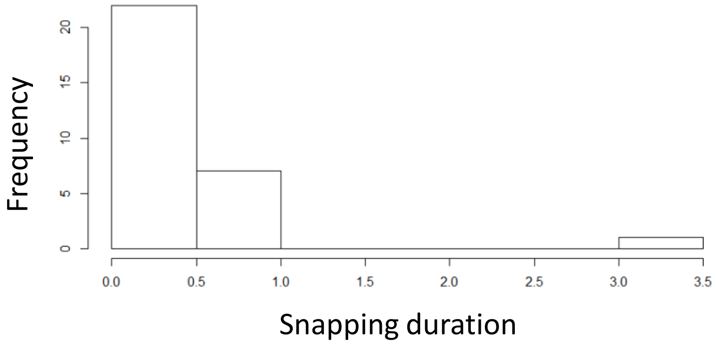

Test of normal distribution

under water (GNU R 3.1.1; Shapiro-Wilk test; *shapiro.test()*-function; *stats*-package)

Trap length

Normal distribution
(W=0.9397; $p > 0.05$)

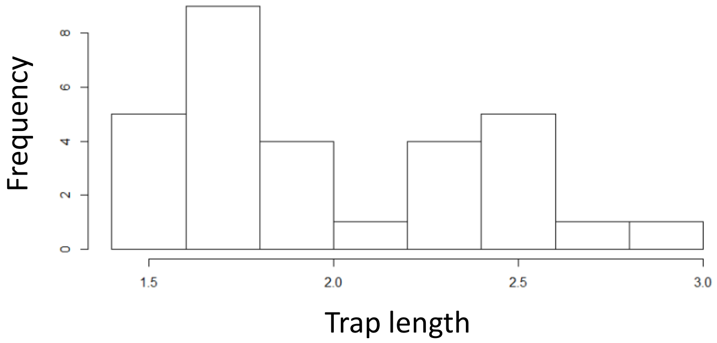

Snapping duration

No normal distribution
(W=0.8329; $p < 0.001$)

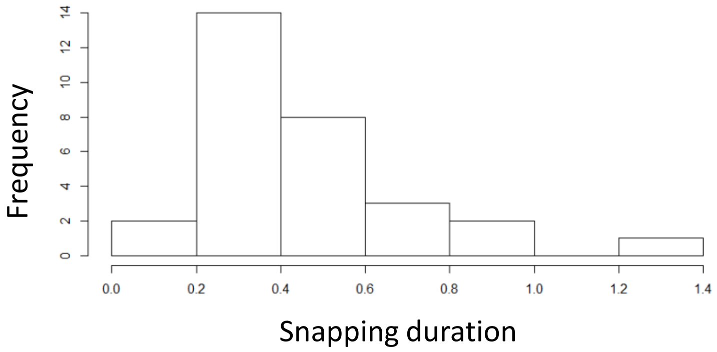

Test of homoscedasticity

Test of homoscedasticity

Trap length (GNU R 3.1.1; LeveneTest; *leveneTest()-f*unction; *car*-package)

Air vs. water	**Homoscedastic**<br>(*df*=[1.58]; *F*=2.4848 ; *p* > 0.05)

Snapping duration (GNU R 3.1.1; LeveneTest; *leveneTest()*-function; *car*-package

Air vs. water	**Homoscedastic**<br>(*df*=[1.58]; *F*=0.1542 ; *p* > 0.05)

Test of significance

Test of significance 1 – Are the trap lengths significantly different between water and air?

GNU R 3.1.1; Wilcoxon rank sum test; wilcox.test*()*-function; stats-package

The trap lengths are not significantly different between the surrounding media!
(W=487.5 ; $p > 0.05$)

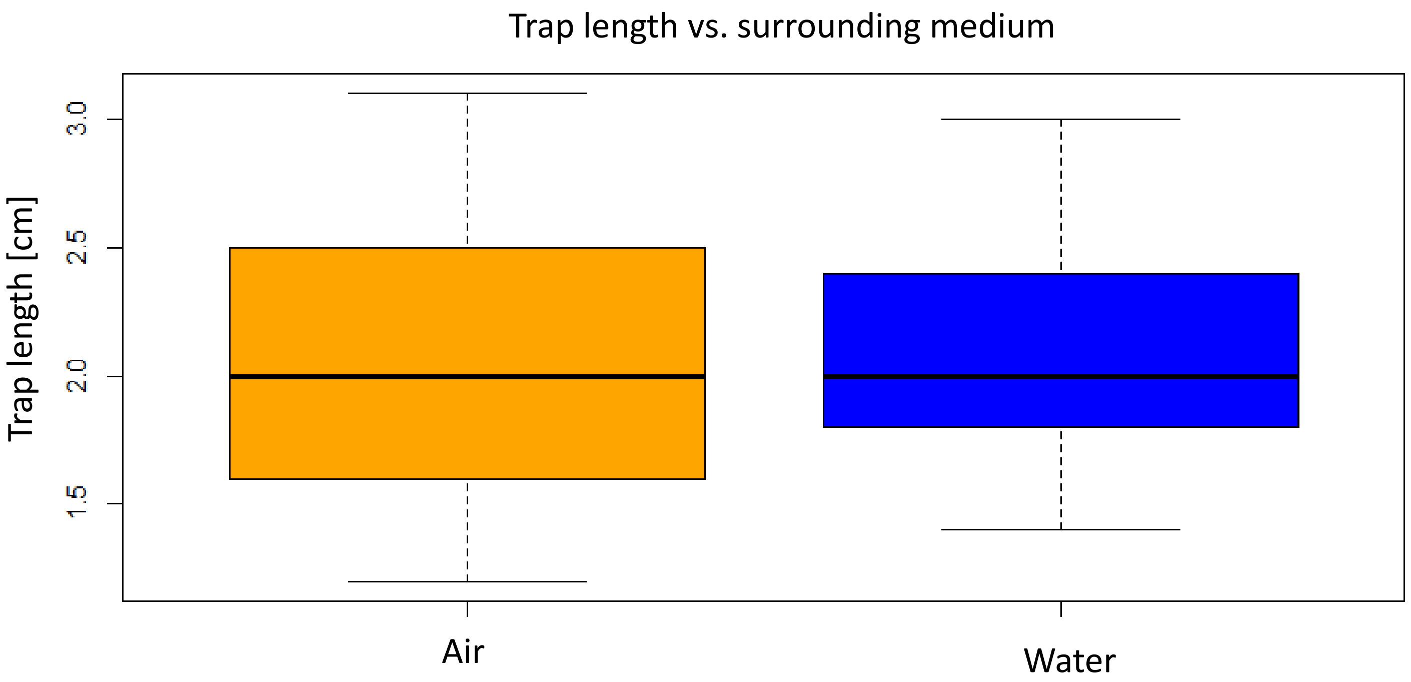

Data can be pooled!

Test of significance 2 – Are the snapping durations significantly different between water and air?

GNU R 3.1.1; Wilcoxon rank sum test; wilcox.test*()-f*unction; stats-package

The snapping durations are not significantly different between the surrounding media!
(W=472 ; $p > 0.05$)

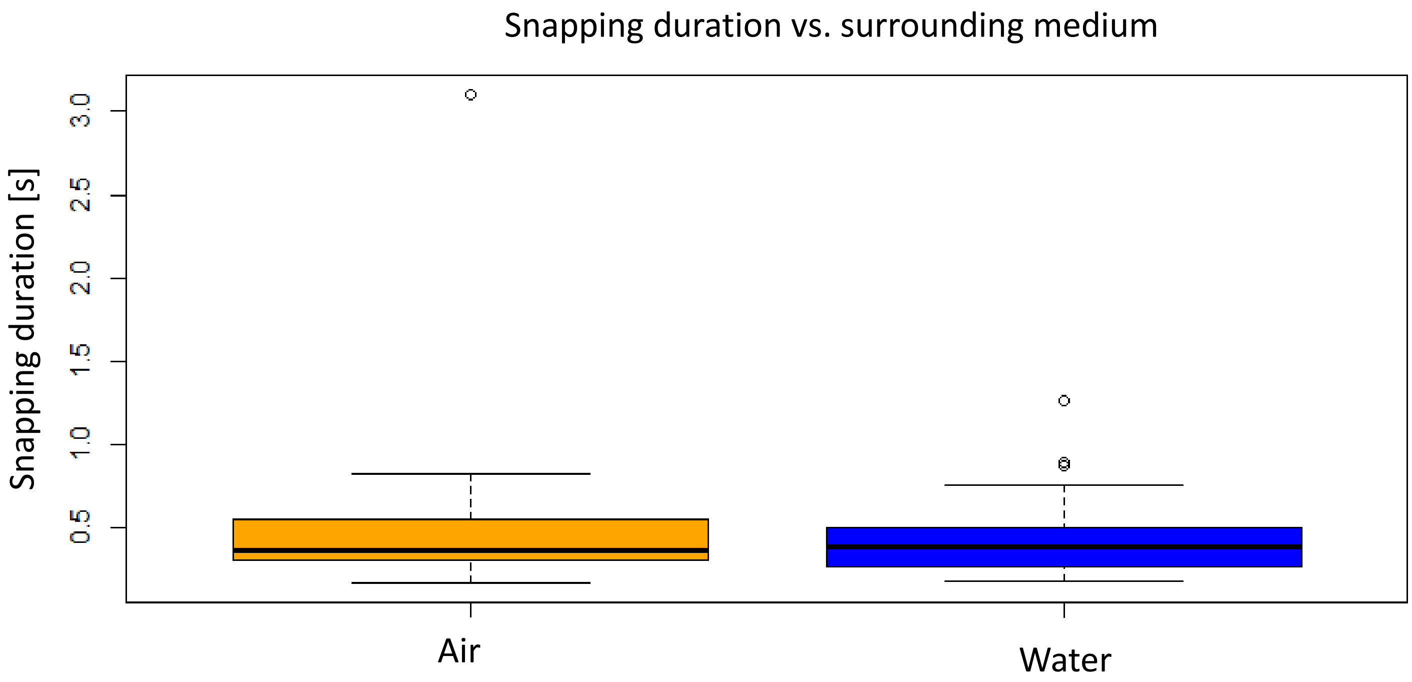

Data can be pooled!

Correlation

Correlation between trap length and snapping duration

GNU R 3.1.1; Spearman correlation (rho); cor*()*-function; stats-package

The snapping durations do not correlate with the trap lengths.
However, there is a weak tendency that larger traps snap faster.
(Spearman‘s *rho* = -0.21)

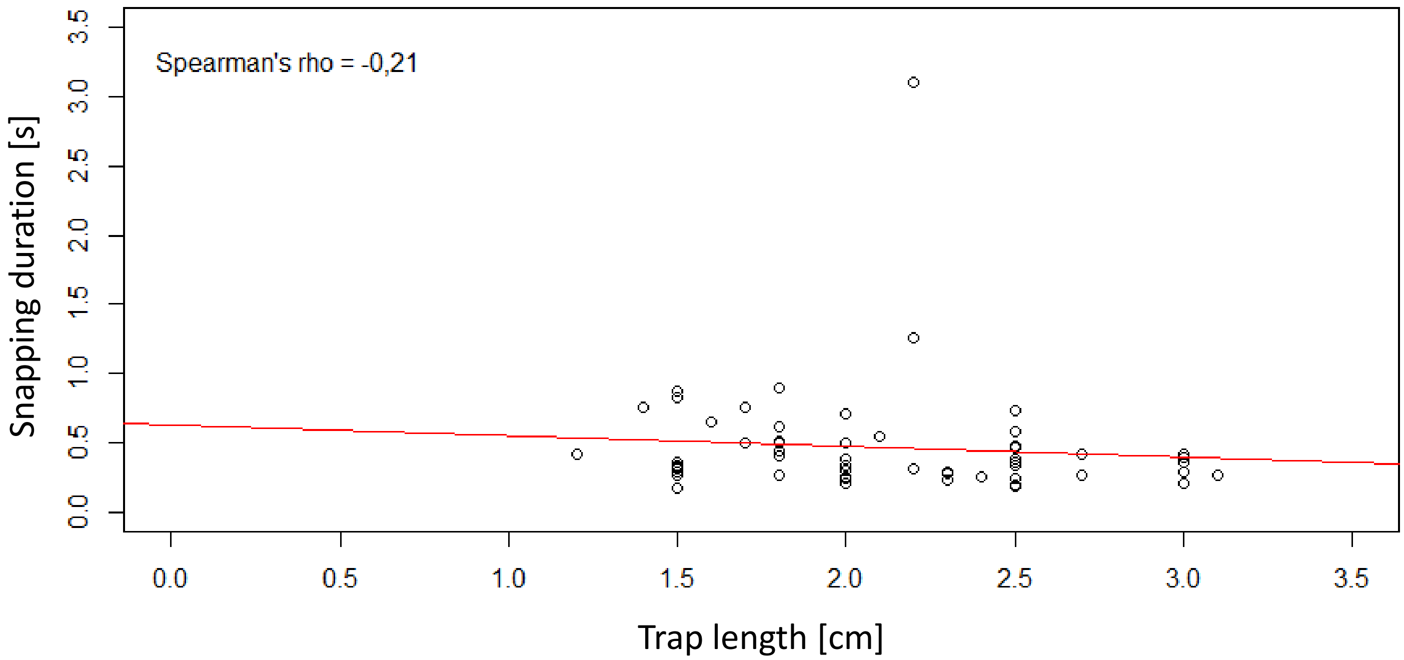

Fisher‘s exact test

Fisher‘s exact test – Are the snapping modes independent of the surrounding media?

GNU R 3.1.1; Fisher‘s exact test; fisher.test*()*-function; stats-package

Contingency table	In air	under water
Synchronous lobe movement	21	18
Asynchronous lobe movement	9	12

Snapping modes are independent of the surrounding media!
($p > 0.05$)

Used packages

- *stats*: Standard package of GNU R. ref. 1
- *car*: ref. 2
- *psych*: ref. 3

References:

1. R Core Team (2014). R: A language and environment for statistical computing. R Foundation for Statistical Computing, Vienna, Austria. URL http://www.R-project.org/.

2. John Fox and Sanford Weisberg (2011). An {R} Companion to Applied Regression. Second Edition. Thousand Oaks CA: Sage. URL:http://socserv.socsci.mcmaster.ca/jfox/Books/Companion

3. Revelle. W. (2015) psych: Procedures for Personality and Psychological Research. Northwestern University, Evanston, Illinois, USA. http://CRAN.R-project.org/package=psych Version = 1.5.4.
