# Supplementary material for: Comparative kinematical analyses of Venus flytrap (Dionaea muscipula) snap traps
Source: Beilstein J Nanotechnol. 2016 May 4;7:664–74. doi: 10.3762/bjnano.7.59 (PMC4902084; doi:10.3762/bjnano.7.59)
Supplement: File 3 — Statistical analysis: Venus flytrap seedlings. [file Beilstein_J_Nanotechnol-07-664-s003.pdf]

Supporting Information

for

Comparative kinematical analyses of Venus flytrap (*Dionaea muscipula*) snap traps

Simon Poppinga*[1,2], Tim Kampowski[1,2], Amélie Metzger[1], Olga Speck[1,2,3] and Thomas Speck[1,2,3]

Address: [1]Plant Biomechanics Group, Botanic Garden, University of Freiburg, Schänzlestrasse 1, 79104 Freiburg im Breisgau, Germany; [2]Freiburg Materials Research Center (FMF), University of Freiburg, Stefan-Meier-Straße 21, 79104 Freiburg im Breisgau, Germany and [3]Freiburg Center for Interactive Materials and Bio-Inspired Technologies (FIT), Georges-Köhler-Allee 105, 79110 Freiburg im Breisgau, Germany

Email: Simon Poppinga* - simon.poppinga@biologie.uni-freiburg.de

*Corresponding author

Statistical analysis: Venus flytrap seedlings

Descriptive statistics

Descriptive statistics

Whole data set

Parameter	Trap length (cm)	Snapping duration (s)
Sample size (n)	12	12
Mean	0.45	10.56
SD	0.07	6.50
Median	0.46	7.63
IQR	0.06	8.61
Min	0.31	4.96
Max	0.53	21.82
Range	0.22	16.86

Test of normal distribution

Test of normal distribution

Whole data set (GNU R 3.1.1; Shapiro-Wilk test; *shapiro.test()*-function; *stats*-package)

Trap length

Normal distribution
(W=0.8846; $p > 0.05$)

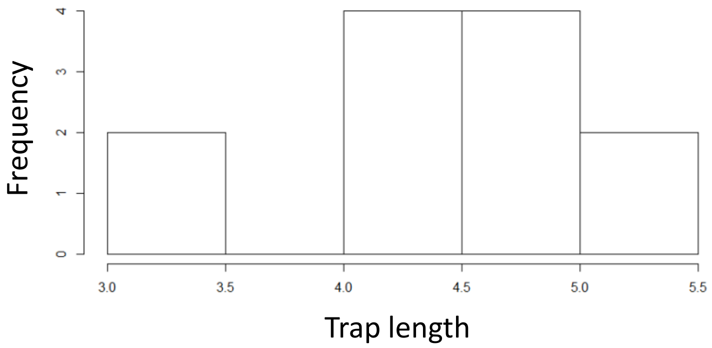

Snapping duration

No normal distribution
(W=0.7859; $p < 0.01$)

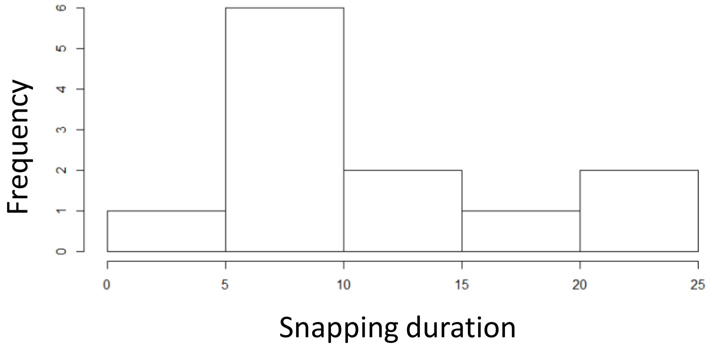

Test of homoscedasticity

Test of homoscedasticity

Trap length (GNU R 3.1.1; LeveneTest; *leveneTest()-f*unction; *car*-package)

Adult vs. seedling	**Heteroscedastic**<br>(*df*=[1.31]; *F*=15.675 ; *p* < 0.001)

Snapping duration (GNU R 3.1.1; LeveneTest; *leveneTest()*-function; *car*-package

Adult vs. seedling	**Not homoscedastic**<br>(*df*=[1.31]; *F*=18.951 ; *p* < 0.001)

Test of significance

Test of significance 1 – Are trap lengths in adults and seedlings significantly different?

GNU R 3.1.1; Wilcoxon rank sum test; wilcox.test*()*-function; stats-package

Trap lengths are highly significantly different!
(W=252 ; $p < 0.001$)

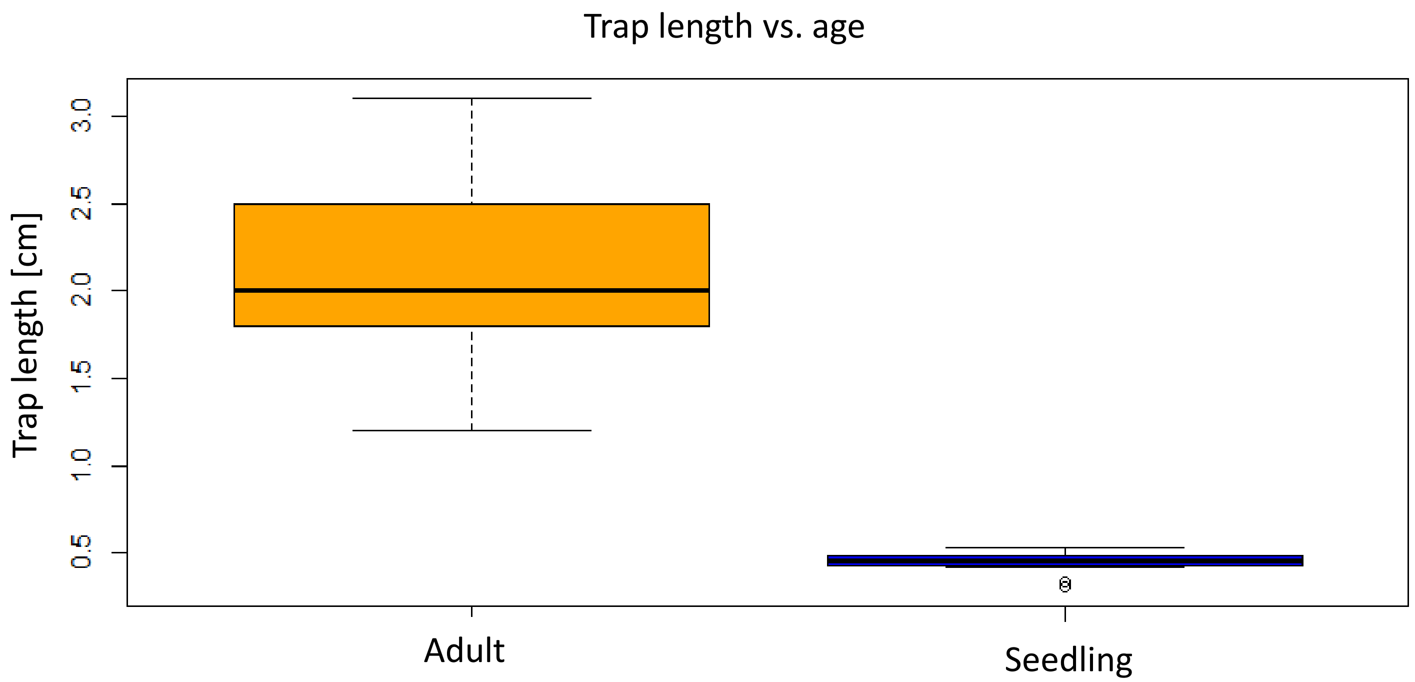

Data set of **adult traps** contains 21 traps used for the comparative air/water snapping experiment which showed synchronously moving lobes
– see results and experimental in main text.

Test of significance 2 – Are snapping durations in adults and seedlings significantly different?

GNU R 3.1.1; Wilcoxon rank sum test; wilcox.test*()*-function; stats-package

Snapping durations are highly significantly different!
(W=0 ; $p < 0.001$)

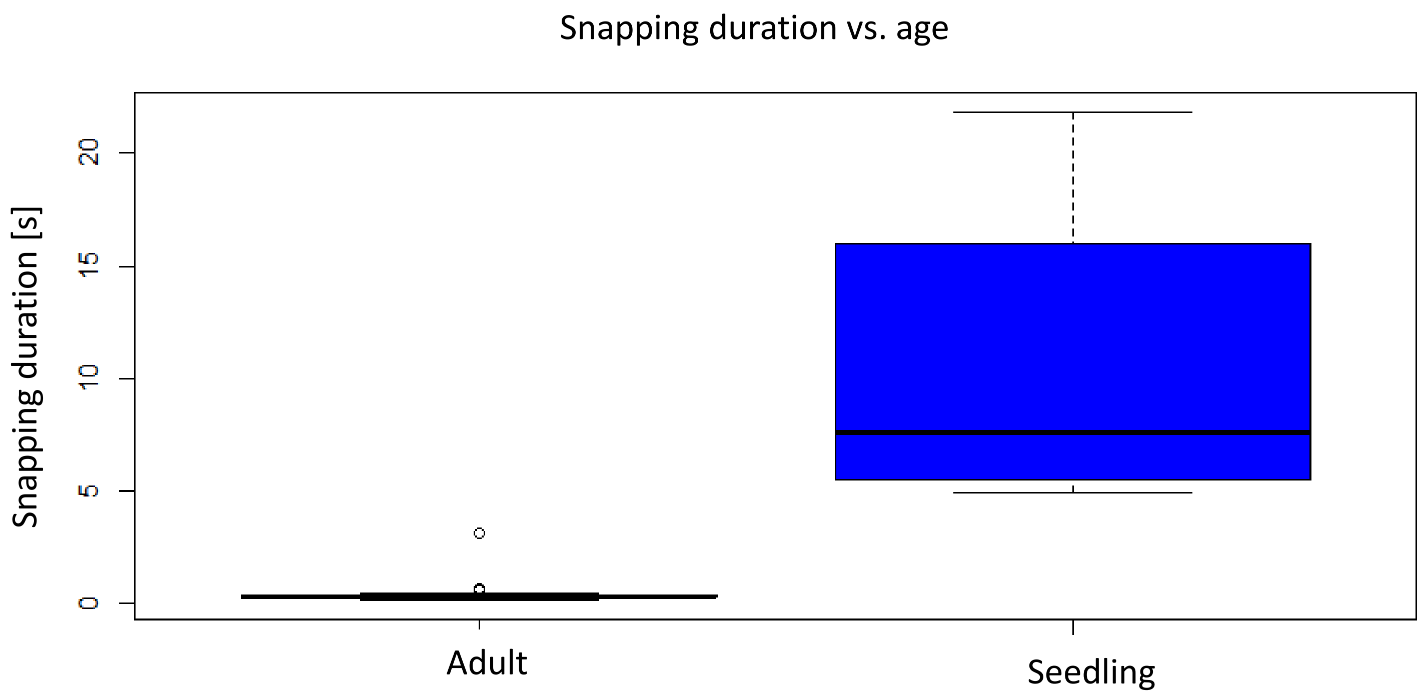

Correlation

Correlation between trap length and snapping duration

GNU R 3.1.1; Spearman correlation (rho); cor*()*-function; stats-package

Snapping durations and trap lengths do not correlate!
(Spearman‘s *rho* = -0.11)

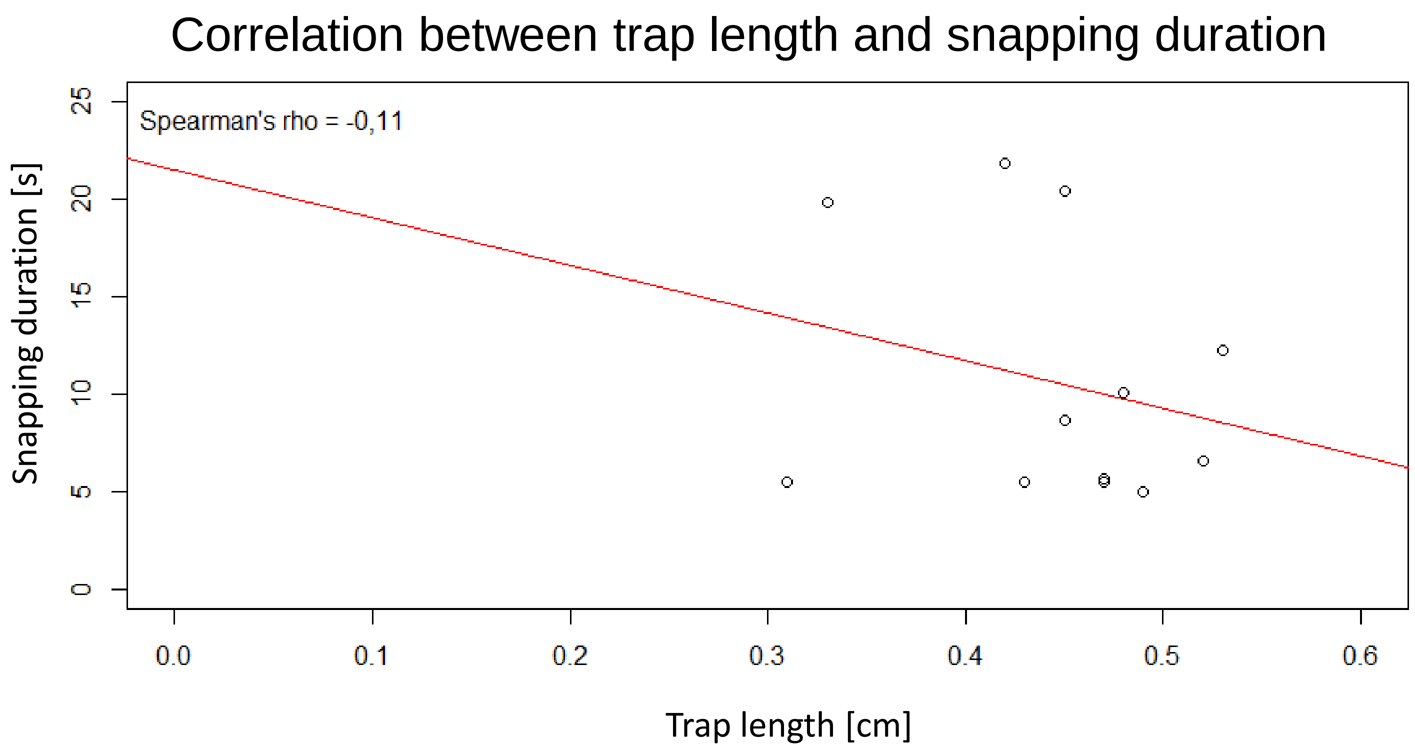

Used packages

- *stats*: Standard package of GNU R. ref. 1
- *car*: ref. 2
- *psych*: ref. 3

References:

1. R Core Team (2014). R: A language and environment for statistical computing. R Foundation for Statistical Computing, Vienna, Austria. URL http://www.R-project.org/.

2. John Fox and Sanford Weisberg (2011). An {R} Companion to Applied Regression. Second Edition. Thousand Oaks CA: Sage. URL:http://socserv.socsci.mcmaster.ca/jfox/Books/Companion

3. Revelle. W. (2015) psych: Procedures for Personality and Psychological Research. Northwestern University, Evanston, Illinois, USA. http://CRAN.R-project.org/package=psych Version = 1.5.4.
